# Case Report: Feline spinous process giant-cell osteosarcoma

**DOI:** 10.3389/fvets.2026.1779067

**Published:** 2026-04-30

**Authors:** Dries K. M. Vercoutere, Lucinda L. Van Stee, Erik A. W. S. Weerts, Björn P. Meij

**Affiliations:** 1 Small Animal Surgery, Department of Clinical Sciences of Companion Animals, Faculty of Veterinary Medicine, Utrecht University, Utrecht, Netherlands; 2 Veterinary Pathology, Department Biomolecular Health Sciences, Faculty of Veterinary Medicine, Utrecht University, Utrecht, Netherlands

**Keywords:** axial osteosarcoma, collision tumor, feline, giant cell osteosarcoma, neurosurgery, spinal surgery, surgical oncology, telangiectatic osteosarcoma

## Abstract

Two adult domestic shorthair cats were evaluated for progressive neurologic deficits caused by spinal cord compression secondary to vertebral osteosarcoma, giant-cell subtype. Feline giant cell osteosarcoma represents a rare tumor type, particularly uncommon in the vertebral column. The cats showed progressive paraparesis or hemiparesis, with neuroanatomic localization to C1–C5 and T3–L3 respectively. Magnetic resonance imaging revealed a single, well-demarcated osseous mass arising from the spinous process for and lamina for each patient (case 1 at C6 and case 2 at T4). Both lesions produced marked >50% dorsal spinal cord compression and were isointense to the spinal cord on T1-weighted images and hypointense on T2–weighted images. Cytology prior to surgical intervention was performed in one case, confirming sarcoma diagnosis, and both affected cats underwent a dorsal laminectomy procedure, involving the removal of the affected spinous process and lamina. Histopathology was performed for both patients and confirmed giant cell osteosarcoma in both cases and additional features consistent with the telangiectatic subtype of osteosarcoma in one case. Post-operative recovery included initial resolving of presenting clinical signs, though both cats ultimately experienced recurrent neurological deterioration consistent with local tumor recurrence, leading to euthanasia. These cases illustrate that spinal giant cell osteosarcoma should be considered in cats with progressive myelopathy. They demonstrate that surgical decompression alone can provide substantial neurological improvement, with the potential for medium-term stability or remission. As local tumor recurrence caused eventual relapse of clinical signs, improving pre-, and intraoperative tumor margin identification and applying adjuvant treatments may improve longterm outcome, and should be investigated in future cases.

## 1 Introduction

Osteosarcoma is an uncommon malignant bone tumor in cats, with most cases arising in the appendicular skeleton (1–3). Axial osteosarcomas are less prevalent, while vertebral involvement is even more rare (1, 2). Feline giant cell osteosarcoma as a subtype is considered a relatively uncommon histologic variant of feline osteosarcoma. Notably, its occurrence within the vertebral column is seldom reported (3–5). Because the feline vertebral canal is anatomically confined, even limited neoplastic expansion can result in clinically significant spinal cord compression and progressive myelopathy (5). The scarcity of reported cases restricts our understanding of the typical clinical presentation, imaging features, surgical feasibility, histopathologic patterns, and long-term prognosis of vertebral osteosarcoma in feline patients. This case report contributes to the limited amount of knowledge regarding this subject, by describing two cats diagnosed with vertebral giant-cell osteosarcoma. The aim is to describe the clinical presentation, imaging characteristics, surgical management, histopathological diagnosis, and clinical outcomes to enhance recognition and decision-making of vertebral giant-cell osteosarcoma.

## 2 Case descriptions

### 2.1 Case 1

A 7 year-old 4.8 kg spayed female domestic shorthair cat presented with a 1.5 month history of progressive gait abnormalities, initially affecting the right thoracic limb and subsequently involving the right pelvic limb. The owner reported reluctancy to jump and a low posture gait. At general physical examination, no abnormalities were found. Prior to referral, the only treatment administered was a daily vitamin B supplement.

Neurological examination revealed a right-sided hemiparesis, more pronounced in the thoracic limb than the pelvic limb. Passive movement of the head to the right produced resistance and mild head rotation, whereas movement to the left was performed readily and maintained within the horizontal plane. Postural reactions were delayed in the right thoracic and pelvic limbs. No further neurologic abnormalities were found. Segmental spinal reflexes in the thoracic limbs were within normal limits, and no lower motor neuron signs were detected. Neuroanatomic localization was therefore most consistent with a C1–C5 spinal cord lesion, based on unilateral upper motor neuron paresis without evidence of thoracic limb hyporeflexia or muscle atrophy. However, low field MRI (0.2 T Magnetom Open Viva, Siemens) of the complete cervical spine (C1–C7) demonstrated a well-demarcated soft-tissue mass arising from the dorsal aspect of the C6 vertebra. The mass involved the spinous process and extended into the dorsal lamina with consequent displacement and dorsal compression of the spinal cord. Given the pre-dominantly dorsal extradural location of the lesion and the apparent absence of lower motor neuron deficits, the clinical presentation may reasonably have mimicked a C1–C5 myelopathy despite the anatomical origin at C6.

Pre-operative radiographs confirmed a localized osseous abnormality affecting the C6 spinous process and dorsal lamina. Thoracic radiographs were acquired to exclude metastatic disease and to plan surgical intervention.

A dorsal laminectomy, centered over C6, was performed through a dorsal midline approach, in which the spinous processes of C5, C6 and C7 were exposed. The spinous processes of C5 and C7 were removed en block for separate histopathological evaluation. The mass associated with C6 was circumferentially dissected and excised, and further decompression of the spinal cord was performed. The resulting osseous defect was filled with an autologous fat graft prior to layered closure. Musculature and fascia were closed using interrupted sutures (3-0 polydioxanone). Subcutaneous tissues were closed using simple continuous suture pattern (3-0 poliglecaprone 25) and the skin was closed using simple interrupted sutures (4-0 Nylon). Post-operative management included cage rest for 6 weeks, meloxicam [0.05mg/kg orally (PO), once daily (SID) for 10 days], and a transdermal fentanyl patch for perioperative pain control.

At the first post-operative recheck at 5 days post-surgery, the owner reported persistent discomfort and episodes of hindlimb hyperextension, after which the transdermal fentanyl patch was replaced. In the following 2 weeks, the owner noted steady improvement: the cat became less painful, gait quality improved and dragging of the pelvic limbs diminished. One month after surgery, the right thoracic limb function improved substantially; with ambulatory and motor function improvement. The patient became more active and regained the ability to climb stairs. Symptoms of ataxia persisted in the follow-up period, but the overall neurological function continued to improve compared to the pre-operative presentation.

In subsequent months, intermittent episodes of instability were reported. At 6 months after surgery, the owner reported a low posture gait and mild generalized weakness. The recommendation to repeat cervical MRI to assess recurrence or instability was advised. At approximately 14 months after surgery, the clinical signs progressed, consistent with those present at initial presentation. Despite our advice for repeat imaging, the owner declined further evaluation as the owner refrained from additional treatment. The cat was euthanized shortly after, and no post-mortem examinations were performed. Tumor recurrence was suspected but not confirmed.

### 2.2 Case 2

A 4-year-old 5.0 kg neutered male domestic shorthair cat was presented with progressive pelvic limb dysfunction for 3 weeks. The owner noted progressive thoracolumbar pain on palpation, reluctance to jump, and frequent collapsing of the pelvic limbs. Weight loss of 0.5 kg within 1 week before referral was noted. Initial assessment by the primary veterinarian revealed no abnormalities and a short course of injectable analgesics produced only partial and transient improvement. Despite unremarkable radiographs at the referring clinic, the cat's gait instability and thoracolumbar discomfort progressed, prompting referral.

The general physical examination was unremarkable. Gait assessment revealed ambulatory paraparesis with marked pelvic limb ataxia and occasional falling to either side. Dorsal extension of the thoracolumbar spine elicited marked pain. Postural reactions were delayed in both pelvic limbs, while segmental spinal reflexes remained normal. The neurological findings were most consistent with a T3–L3 neuroanatomic localization.

**Figure 1:**
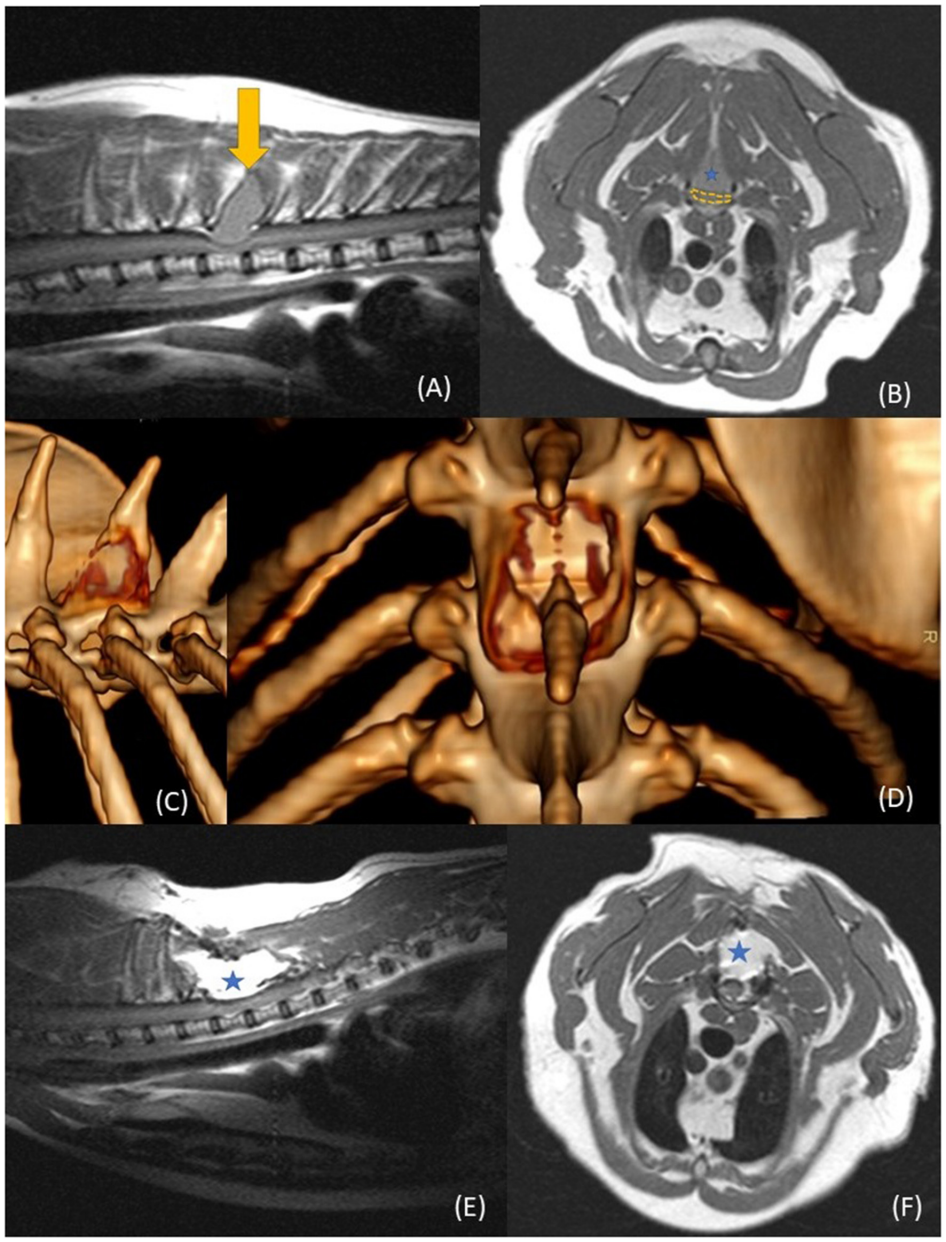
Case 2 pre-operative and post-operative imaging. **(A)** pre-operative sagittal T1 weighted MRI image with a mass (yellow arrow) at the level of the T4 vertebra. **(B)** pre-operative transverse T1 weighted MRI image with a mass (blue star) at the level of the T4 vertebra. The mass bulges ventrally into the spinal canal, compressing the spinal cord (yellow dotted line). **(C)** A lateral and **(D)** dorsal view of the 3D rendering of the pre-operative CT images. The mass in the spinous process shows significant osteolysis and as an expansive behavior. **(E)** T1 weighted sagittal and transverse **(F)** MRI images at 6 months post-surgery. There is hyperintense tissue mass present (blue star), marking the autologous fat graft placement in the laminectomy defect.

MRI (0.2 T Magnetom Open Viva, Siemens) of the thoracic spine was performed and revealed a 1.2 x 1.7 cm expansile osseous mass originating from the spinous process of T4 (Figures 1A, B). The mass extended into the dorsal lamina and caused a severe (>50%) dorsal spinal cord compression. The lesion was isointense to the spinal cord on T1-weighted images and hypointense on T2-weighted images, with a thin cortical rim surrounding the mass.

Computed tomography (CT), performed on the same day, confirmed that the lesion originated from the T4 spinous process (Figures 1C, D). During CT, a fine-needle aspiration was obtained and revealed spindle-shaped mesenchymal cells and multinucleated giant cells.

Surgical decompression in combination with tumor excision was performed through a dorsal approach, extending from T1 to T6. The spinous processes of T3 to T5 were removed en block for histological examination. The dorsal laminectomy was subsequently carried out from T3 caudally to T5 cranially for decompression of the spinal cord. A bilateral hemilaminectomy through T4 allowed resection of the T4 spinous process together with the associated mass en bloc. The tumor consisted of a thin cortical shell enclosing the neoplastic tissue. Following excision, an autologous fat graft obtained from subcutaneous tissue was placed over the defect before routine closure as described in case 1.

Post-operative management included cage rest and multimodal analgesia of fentanyl (5 μg/kg/h) and ketamine (5 μg/kg/min) continuous-rate infusions (CRI), meloxicam (0.05 mg/kg SID IV), buprenorphine (20 μg/kg TID IV). Post-operative intermittent tachycardia and suspected hypovolemia were managed with intravenous fluid boluses. Cardiovascular monitoring continued overnight, and echocardiography on the day after surgery showed no structural abnormalities. The cat was discharged on 2 days after surgery with continued analgesia and instructions for activity management.

At the 6-week post-operative recheck, the cat showed notable improvement: displaying only mild pelvic limb ataxia without spinal hyperesthesia. The surgical site had healed appropriately, and activity gradually improved. A follow-up MRI was advised within 3 to 4 months. Supplementary chemotherapy was discussed with the oncology department but was not recommended at that time. A repeat MRI performed 6 months after surgery (Figures 1E, F) showed no contrast enhancement, mass effect or spinal cord compression. No evidence of tumor regrowth was detected. At this time, the owner reported an excellent quality of life, with normal activity levels and only occasional mild ataxia. Around 1 year post surgery, the owner reported recurrence of pelvic limb weakness, progressive ataxia, and a rapid decline in hindlimb function. In view of the progressive neurologic deterioration and declining clinical condition, the cat was euthanized 373 days after surgery. No post-mortem examination was performed. A timeline has been constructed for both cases and is available in the section of Supplementary material.

## 3 Histopathology

**Figure 2:**
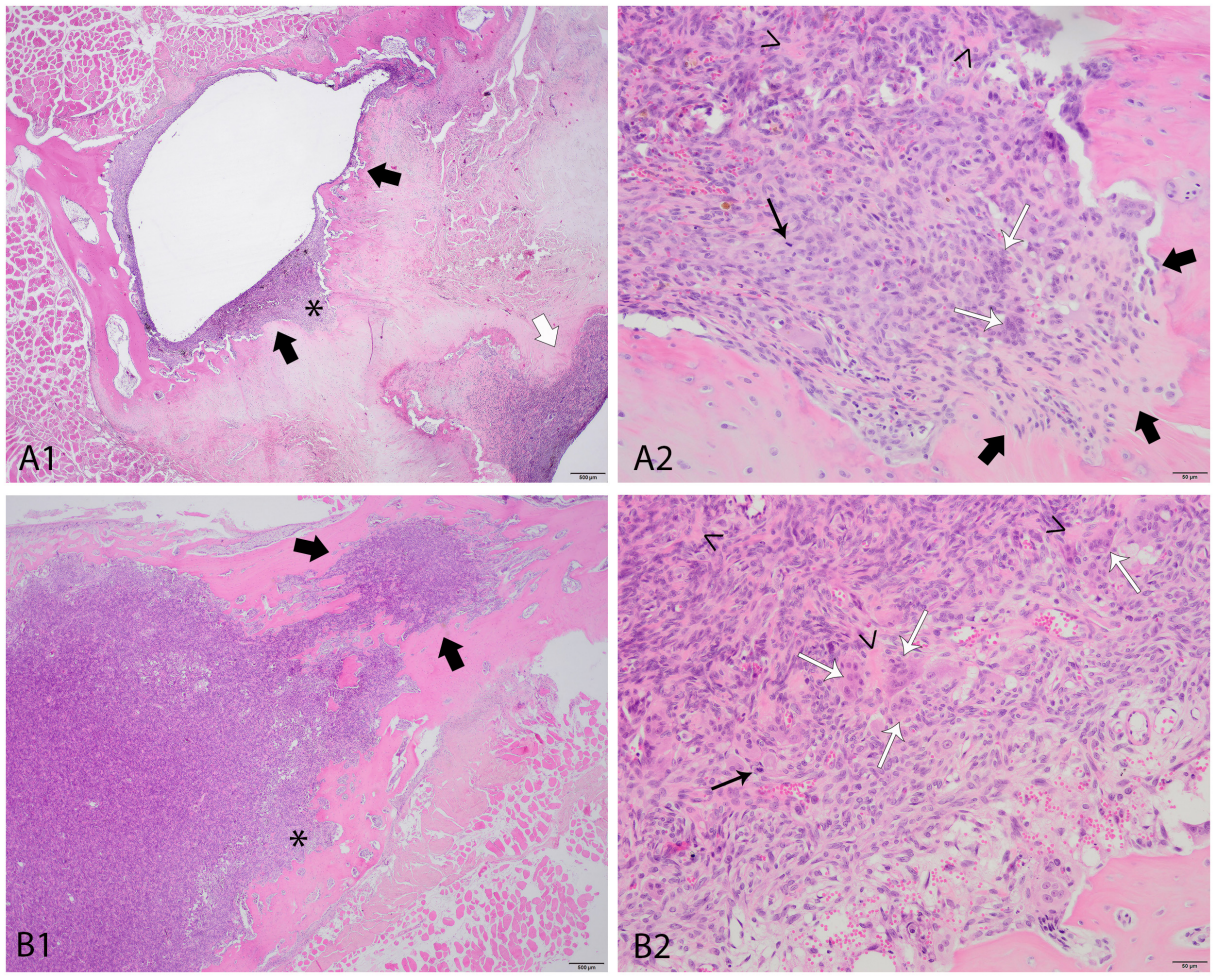
Histopathological photos of the tissue proliferations of both cases, panels **(A)** case 1, panels **(B)** case 2, H&E stain. **(A1)** case 1, low magnification of the affected vertebral bone with adjacent skeletal muscle, the proliferation pointed out with the arrows - white arrow: part adjacent to the vertebral canal - black arrows: part of the proliferation within the spinous process with clear signs of invasive growth, within this part a cystic dilated area is present. *: area magnified in panel **A2**. **(A2)** case 1, high magnification of the proliferation–thick black arrows: area with clear invasive growth–thin black arrow: mitotic figure–thin white arrows: multinucleated cells–arrowheads: small amounts of osteoid. **(B1)** case 2, low magnification the the affected vertebral bone with adjacent skeletal muscle–black arrows: part with clear invasive growth within the pre-existing bone matrix. *: area magnified in panel **B2**. **(B2)** case 1, high magnification of the proliferation–thin black arrow: mitotic figure–thin white arrows: multinucleated cells–arrowsheads: small amounts of osteoid.

After adequate decalcification of the tissue via exposure to acids, histopathologic examination of the excised vertebral lesions from both cats revealed neoplastic proliferative growth with features consistent with giant-cell osteosarcoma (Figure 2). The neoplastic tissue in both cases was composed of interwoven bundles of mildly to moderately pleomorphic spindle-shaped cells with areas that contained numerous multinucleated giant cells. Irregular deposits of osteoid were present between the neoplastic cells, confirming osteoblastic origin. The cells exhibited moderate anisocytosis and anisokaryosis with incidentally prominent nucleoli. In both cases mitotic figures were found, with respectively three and six mitoses counted per 2.37 mm^2^ in cases 1 and 2 In case 1, few cystic areas partially filled with blood were observed within parts of the proliferation (Figure 2A1), which seems to indicate that the proliferation in case 1 also presents with features consistent with the telangiectatic subtype of osteosarcoma. In both cases, the neoplasm arose from the medullary part of vertebral bone with presence of a thin rim of cortical bone that surrounded the neoplastic tissue. In both cases, areas with clear invasive growth within pre-existing bone matrix were observed The combination of biological behavior and morphological features did not seem to fit well with diagnoses of respectively osteoblastoma and so-called ‘aggressive osteoblastoma' as recognized within human classification systems of bone neoplasia.

## 4 Discussion

The two cases presented here illustrate a rare manifestation of feline (giant cell) osteosarcoma affecting the vertebral column. Although osteosarcoma in cats is generally considered less aggressive than in dogs, axial locations, and in particular vertebral lesions can cause severe dysfunction and pain. Even limited tumor expansion can cause substantial spinal compression and progressive neurological deficits as a result (5). In these cases, advanced imaging (MRI/CT) revealed a well-demarcated osseous mass arising from the spinous process and dorsal lamina, producing marked (>50%) dorsal spinal cord compression. The osteosarcoma lesions caused a monostotic, severe deformation of the spinous process and lysis of the osseous structures; all diagnostic imaging characteristics strongly supporting a primary vertebral bone tumor rather than a soft-tissue or intradural lesion. Multimodal imaging–most commonly MRI with CT or radiographs–is strongly recommended for distinguishing extradural osseous tumors from other causes of spinal cord compression (6, 7). MRI revealed a well-defined osseous mass with a isointense T1- and hypointense T2- weighted signal. In case 2, CT-imaging confirmed the expansion of the spinous process and adjacent dorsal lamina. This combination of MRI/CT imaging matches previous reports of vertebral osteosarcoma in cats, in which extradural osseous masses often appear T1-isotense, T2-hypointense (5). Furthermore, cortical expansion has been seen as a main diagnostic tool to define osteosarcomas (7).

Preoperative cytology was performed on case 2, revealing pleomorphic mesenchymal cells and multinucleated giant cells, features compatible with an osteogenic neoplasm. Although definitive diagnosis of osteosarcoma relies on histopathologic evaluation, cytologic assessment provides minimally invasive preliminary information (8, 9). Especially as other causes of spinal masses can include round cell neoplasia, which may not have a surgical indication. In this context, the cytologic findings in case 2 demonstrate that pre-operative cytology may serve as an integral part in future diagnostic work-up of spinal masses. Conclusively, by combining multimodal imaging and cytologic assessments in these cases, we were able to concisely validate the diagnosis of these vertebral lesions and develop an individualized treatment plan.

Surgical decompression was performed in both cats as a palliative treatment option to relieve spinal cord compression. This resulted in a short-term neurological improvement by restoring ambulation and comfort. Though the results did not sustain over time, the clinical relief that was achieved was highly valued by owners. Similar to reports in other feline vertebral osteosarcoma cases, surgical decompression thus provides symptomatic relief and is often chosen as an initial treatment option (5, 10). However, because laminectomy primarily alleviates mass effect and the location prohibits complete tumor resection, recurrence is likely when wide margins cannot be obtained (9). More invasive procedures, such as vertebrectomy with stabilization, may offer longer survival in selected cases by enabling more complete tumor excision. These procedures, however, are technically demanding, expensive, and not widely available (11). In many cases, decompressive debulking therefore represents an acceptable palliative treatment option, even when long-term tumor control cannot be achieved. Both cases encountered recurrent clinical signs. The most likely cause would be recurrent disease. Either due to local dorsal regrowth at the surgical site, progression of initial microscopic residual disease extending into the vertebral body, or potential intraoperative neoplastic cell seeding (12). Alternatively, local spinal column instability, created during the surgical procedure, could cause deterioration (13). In human medicine, epidural fibrosis after spinal decompressive surgery is mentioned as a possible cause of treatment failure (14), where dogs are used as a model of disease, however, this has not been reported in cats nor dogs in a clinical setting. The precise nature of the cause of clinical decline in the present cases could not be definitively determined, as no repeat imaging at the time of deterioration or post-mortem examination was performed. Although local tumor recurrence, either by remnant tumor tissue not visible at the time of surgery, or tumor seeding during dissection, alternative mechanisms cannot be excluded. In the absence of confirmatory imaging or necropsy findings, the exact pattern of recurrence remains speculative and should be interpreted with caution. Consequently, while more extensive surgical approaches may theoretically improve local control, the present cases do not allow definitive conclusions regarding the anatomical nor pathological source of recurrence of clinical signs.

Reports of cases managed with incomplete or marginal excision, local regrowth is expected within several weeks to a few months (9, 11). The time to recurrence in case 1 falls within this expected window (7 months until clinical deterioration), whereas the approximately 1-year recurrence observed in case 2 represents the longer end of the anticipated interval. In contrast, reports describing the combination of local surgery and chemotherapy have documented substantially longer periods of disease control (10). For example, vertebral osteosarcoma case with complete remission at 16 months post-operatively or two cats with vertebral osteosarcoma showing excellent short- to medium-term outcomes up to 28 months (5). Conclusively, our cases support the view that decompressive surgery alone is primarily palliative while multimodal protocols may prolong remission in selected patients. Adjunctive treatments–including radiotherapy, chemotherapy or targeted agents–may help prolong remission when the primary tumor has been debulked through surgical intervention (10). Therefore, multimodal strategies may offer a more durable degree of long-term tumor control in selected cases compared with decompression alone. If initial adjunctive treatment options are not available, or declined, regular post-operative repeat imaging may identify tumor recurrence in an earlier stage. Recognizing relapse based on clinical signs alone may increase patient morbidity as signs may not be recognized in a timely manner. Relapse in an early stage may also be more amenable for treatment attempts when compared to late stage disease.

While prolonged remission has been reported, it remains important to recognize that the principal challenge in feline osteosarcoma is local recurrence rather than metastatic spread. Feline osteosarcoma metastasizes far less frequently than in canines (9), suggesting that it is unlikely that systemic chemotherapy alters prognosis with incomplete primary tumor resection. Furthermore, conventional radiotherapy near the spinal cord carries a recognized risk of radiation-induced myelopathy and demyelination in cats and dogs, which may limit its clinical application in this anatomical region (15, 16). However, most available data are derived from conventional fractionated protocols, and information regarding the safety and efficacy of stereotactic radiotherapy in feline spinal tumors remains limited (17, 18). As advanced radiotherapy techniques continue to evolve in veterinary medicine, their potential role in improving local tumor control while minimizing spinal cord toxicity warrants further investigation. This may partly explain why long-term survivors in reported literature most often underwent more extensive or effective local excision rather than deriving an advantage from adjuvant therapy (5, 10, 11). Future progress may therefore rely on achieving more complete surgical resection, supported by enhanced pre- and peri-operative visualization, such as ICG/fluorescence-guided techniques, and high-resolution pre-operative imaging to optimize margin assessment and to ensure the maximal tumor excision possible. While these studies are derived from neurosurgical literature, these studies provide principle-based support for our concept that enhanced visualization and imaging guidance can facilitate a more complete tumor resection (19–21).

## 5 Limitations, future, and conclusion

The outcomes in these two cats show that decompressive surgery can restore neurological function in the short term, but long-term tumor control remains difficult when complete excision is not possible. The recurrence timelines of our cases are broadly consistent with the recurrence window described in the literature and nuance the variable biological behavior of vertebral osteosarcoma.

Several important limitations must be acknowledged. First, diagnostic imaging at the time of clinical deterioration was not performed in either case. As a result, objective confirmation of tumor recurrence or other causes including iatrogenic spinal instability, through repeat MRI or CT was absent. Second, no post-mortem examination was performed in either cat, precluding definitive histopathological confirmation of possible recurrent disease. Consequently, although tumor recurrence remains the most plausible explanation for the observed neurological decline, this assumption cannot be positively affirmed. Alternative explanations, including post-operative dural fibrosis, instability, or other progressive spinal pathology, can therefore not be entirely excluded. These limitations temper the strength of our conclusions and restrict definitive statements regarding recurrence patterns and tumor pathophysiology in these cases.

Looking forward, improved outcomes may depend on achieving more complete local tumor control, supported by advances in pre- and peri-operative imaging and visualization techniques to maximize the extent of safe tumor excision. Adjunctive treatments can be considered to complement the palliative benefits of decompressive surgery in selected cases. Ultimately, these findings emphasize the importance of setting realistic expectations with owners and adjusting treatment plans to prioritize feasibility, neurological function, and quality of life.

## Data Availability

Raw data not presented in this manuscript on the two described cases are available upon request to the authors.

## Supplementary material

Supplementary Figure 1 Timeline. The figure illustrates the chronological timeline of clinical events for the two cases: case 1 (C6) in blue and case 2 (T4) in green. Each timestamp represents a relevant event, including symptom onset, diagnostic imaging (MRI, magnetic resonance imaging; CT, computed tomography; FNA, fine-needle aspiration), surgery, post-operative evaluations, follow-up imaging, recurrence, and outcome. The timeline is semi-proportional, meaning that distances between data points do not precisely represent absolute time intervals but give insight in relative chronological order.
