# Supplementary figures and images for: Case Report: Feline spinous process giant-cell osteosarcoma
Source: Front Vet Sci. 2026 Apr 30;13:1779067. doi: 10.3389/fvets.2026.1779067 (PMC13173541; doi:10.3389/fvets.2026.1779067)

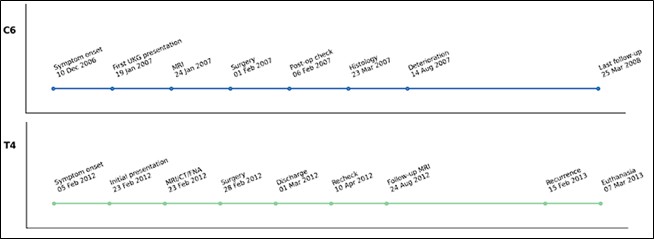

Supplement: Supplementary Figure 1 [file Image_1.jpeg]
